# Microfluidic Laser-Induced Nucleation of Air Microbubbles and Crystals in Urea–Isopropanol Solutions

**DOI:** 10.1021/acs.cgd.5c01702

**Published:** 2026-04-03

**Authors:** Kelechi F. Ndukwe-Ajala, Pierce Haider, Jasmin M. Sabirin, Jiamu Guo, Bruce A. Garetz, Ryan L. Hartman

**Affiliations:** Department of Chemical and Biomolecular Engineering, NYU Tandon School of Engineering, Brooklyn, New York 11201, United States

## Abstract

The conditions for the laser-induced nucleation of urea in isopropanol were investigated. A capillary-based microfluidic system was used to expose supersaturated urea–isopropanol to nanosecond pulses of 1064 nm light. We determined the critical supersaturation of the system to be *S* = 2.2 (equivalent to 6.4 g of urea per 100 g of isopropanol with respect to saturation at 21.5 °C), where crystals spontaneously nucleated in flow and needle crystals were observed. Smaller aspect-ratio prismoid crystals were observed after irradiation of supersaturated solutions with a supersaturation of *S* = 1.65. We noted that a few microbubbles were moving with the crystals and found that microbubbles can be readily generated in the urea–isopropanol solutions by the addition of iron­(II, III) oxide nanoparticles. In flow experiments, the bubbles had an average diameter of 19 μm. Using an inline degasser, we were able to suppress the formation of bubbles, confirming that they are formed from dissolved air in the solution. Needle crystals were observed to transform into smaller aspect-ratio prismoid crystals as the solution warmed to room temperature (21.5 °C). This suggests that the needle crystals are the kinetically favored habit at high supersaturations in urea–isopropanol solutions. We discuss our results in the context of the impurity-heating mechanism for nonphotochemical laser-induced nucleation (NPLIN). The findings presented here provide additional support for the impurity-heating mechanism of NPLIN.

## Graphical abstract

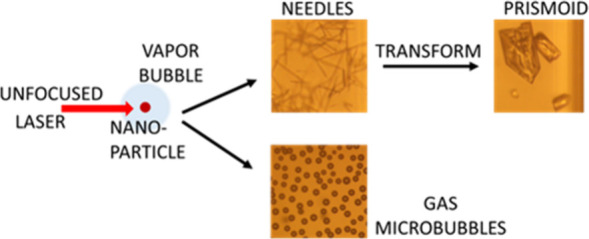

## 1 Introduction

Crystallization is a routine and vital step employed in the refinement and production of materials and chemicals. Controlling crystallization is a useful goal for a broad cross-section of industries aiming for high-quality control and sustainable production. Nucleation refers to the birth of a new phase in which small nuclei emerge from a metastable phase.^1^ For solution crystallization, nascent solid crystals emerge from a supersaturated solution. Nonphotochemical laser-induced nucleation (NPLIN), first demonstrated by Garetz et al.^2^ with supersaturated aqueous urea solutions, is a technique in which solutions are exposed to unfocused nanosecond pulses of light, of wavelengths in the visible and near-infrared region, which accelerate the nucleation of solutes.

Three mechanisms have been postulated: the optical Kerr effect (OKE), the dielectric polarization (DP) model, and the impurity-heating (IH) mechanism. Garetz et al.^2^ proposed that urea solute molecules in prenucleating clusters were aligning with the direction of the electric field through the optical Kerr effect. However, Liu et al.^3^ showed through high-speed digital imaging post laser-induced nucleation that there is no significant correlation between crystal angle and the direction of the plane of polarization of the linearly polarized light. In addition, estimations from molecular simulations by Knott et al.^4^ showed that the interaction energies between the electric field and the urea molecules were too low to account for the accelerated rate of nucleation. The DP model, later proposed by Alexander and Camp, posits that a precritical solute cluster in the presence of an electric field will have a lower activation barrier and become a critical size for nucleation.^5^ The DP model has been used to explain the NPLIN of ionic salts such as potassium chloride^5^ and potassium bromide.^6^ However, the laser-induced nucleation of carbon dioxide bubbles from carbonated water^7^ could not be explained by either the OKE or DP model. Recent studies^8–10^ highlight the contribution of inherent impurity particles present in the solution. Nanofiltration suppressed laser-induced nucleation of aqueous glycine solutions^8^ and aqueous potassium chloride solutions.^9^ The intentional addition of iron oxide nanoparticles reversed the effect of filtration on nucleation yield of aqueous ammonium chloride solutions.^10^ The IH mechanism assumes that nanoimpurities strongly absorb laser energy; the heated nanoparticle transfers energy to the surrounding solution, vaporizing the solvent, and forming a bubble with crystal nucleation occurring at the bubble-solution interface. For aqueous urea, the effect of different impurity microparticles was investigated.^11^ More recently, Barber and Alexander^12^ reported the direct observation of thermocavitation events during NPLIN in aqueous cesium chloride using high-speed imaging and noted that crystals grew at the locations of cavitation events. Ndukwe-Ajala et al.^13^ showed with pressure-dependent studies of NPLIN in aqueous potassium chloride that high pressure suppresses crystal nucleation, which is consistent with bubble suppression.

Two NPLIN studies have been conducted with a solute–alcohol systems. Ikni et al.^14^ investigated laser-induced nucleation of carbamazepine in organic solvents such as methanol and acetonitrile and reported two polymorphs: phases I and III. Li et al.^15^ studied the nucleation of sulfathiazole in water/ethanol mixtures. Both studies also demonstrated the effect of laser polarization on the nucleation of different polymorphs.

Utilizing microfluidics for laser-induced nucleation studies offers several advantages compared to batch glass vials, such as minimal contamination, reduced footprint, safer operation, ideal heat and mass transfer, and high-throughput experimentation. Since 2019, microfluidics has been used to investigate different solute–solvent systems and probe the laser-induced nucleation phenomenon. Hua et al.^16^ demonstrated microfluidic NPLIN for the first time with aqueous potassium chloride solutions and observed that the number of crystals nucleated increased with supersaturation and laser power density and was independent of the number of laser pulses. Hua et al.^17^ also studied the NPLIN of aqueous glycine solutions and developed a model that combined the dielectric polarization model and two-step nucleation theory. Ndukwe-Ajala et al. investigated the effect of iron­(II, III) oxide nanoparticles^18^ and system pressure^13^ on aqueous potassium chloride NPLIN with a single-phase flow system. They observed an increase in nucleation yield with an increasing concentration of intentionally added light-absorbing impurities and an inverse relationship between crystal numbers and system pressure. Korede et al.^19^ developed a droplet-based microfluidic platform combined with a deep learning method for the analysis of droplets with crystals.

A few NPLIN studies^2,3,11,20^ have been conducted with urea solutions. Urea is a model solute for NPLIN due to its fast nucleation kinetics, typically on the order of seconds.^2^ All reported studies thus far have been performed with aqueous urea solutions in glass vials. To our knowledge, the present work is the first to investigate the laser-induced nucleation of urea crystals in an alcohol or an organic solvent. Urea crystals grown in isopropanol were also desirable, as the reported aspect ratio^21^ of such crystals was close to 1, making the system a good candidate for flow NPLIN studies compared to typical needle urea crystals grown in water, which would present flowability challenges. In this work, we explored the operating parameters for laser-induced nucleation of urea–isopropanol solutions. We report the observation of microbubble formation associated with nanoparticle heating and the unexpected nucleation of needle urea crystals in isopropanol.

## 2 Experimental Methods

### 2.1 Materials

Urea (99% ACS grade, Sigma-Aldrich) was dissolved in isopropanol (99% ACS grade, Fisher Scientific) in Schott glass bottles and 4 mL glass vials. Iron­(II, III) oxide nanoparticles dispersed in isopropanol (20 wt % dispersion, particle diameter = 15–20 nm, US Research Nanomaterials Inc.) were used to intentionally dope the filtered urea-isopropanol solutions.

### 2.2 Solution Preparation

Supersaturated solutions were prepared based on the reported saturation concentrations by Lee and Lahti.^22^ Solubility values for intermediate temperatures were approximated using a polynomial fit to the reported solubilities. For instance, at 21.5 °C, the estimated saturation concentration is 2.9 g urea per 100 g isopropanol. Supersaturated solutions were prepared in clean, dry glass bottles. The glass bottles and screw caps used were thoroughly washed with a soap solution (Micro-90) and rinsed at least three times with copious amounts of ultrapure deionized water and then dried in an oven. Measured amounts of urea and isopropanol were transferred into bottles, a magnetic stirrer bar was added to each bottle, and each bottle was placed on a hot plate-stirrer for heating and dissolution. Once dissolved, the bottles were transferred to a forced-air convection oven (Heratherm, Thermo Scientific) set at 60 °C for overnight incubation (>12 h).

### 2.3 Solutions Doped with Nanoparticles

The purchased nanoparticle dispersion was diluted with isopropanol to prepare 2 stock concentrations: 0.44 and 4.4 mg/mL. The concentration was validated by the gravimetric method. Urea–isopropanol solutions were filtered with 220 nm-pore nylon syringe filters into clean glass bottles. Volumes of the iron oxide nanoparticle dispersion (0.5–10 μL) were added to 50 mL filtered solutions. We refer to the addition of nanoparticles to the solutions as "doping". The glass bottle was ultrasonicated for 1 h at room temperature to ensure a uniform dispersion. All doped solutions were inspected by the eye to ensure no spontaneous nucleation had occurred.

### 2.4 NPLIN Flow Experiment

At the core of the system, a square glass capillary contained the flowing solution, which was continuously exposed to the laser, as shown in Figure 1.

**Figure 1:**
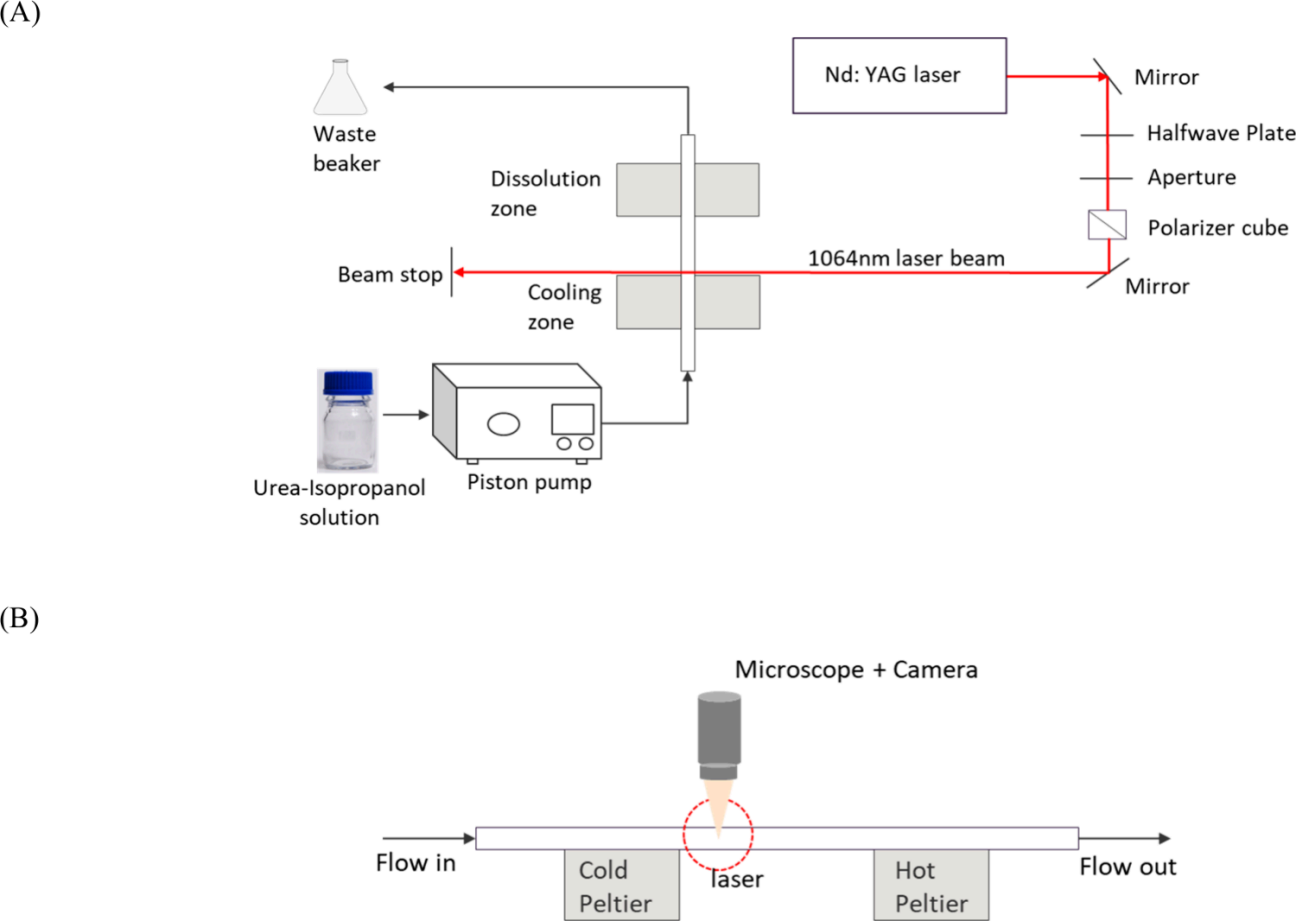
(A) Sketch of the experimental setup. (B) Side view of the flow path showing the observation location above the irradiation zone.

A piston reciprocating pump (LS005SRX, Teledyne Instruments) was used to deliver fluid from the Schott glass bottle at a flow rate of 300 μL/min. The feed bottle was placed on a hot plate-stirrer set to provide a liquid temperature of 40 °C at 100 rpm to keep the solution mixed and warm and to avoid spontaneous nucleation during the flow experiments. Cylindrical polyether ether ketone (PEEK) tubing with a 500 μm ID, 1/16 in. OD was used to connect the pump outlet to the PEEK tubing of 0.020 in. ID, 1/32 in. OD using microtight adapters. Connector sleeves made of clear polycarbonate resin were 3D printed to connect the cylindrical end of the smaller PEEK tubing to the square end of the glass capillary. Clear epoxy (EA E-05CL, Loctite) was used to adhere the smaller PEEK tubing to the square-cross-sectioned borosilicate glass capillary, which had a 1 mm × 1 mm ID, 0.2 mm wall thickness, and 100 mm length (8100, VitroCom).

The solution temperature before irradiation was further cooled by contact of the capillary with a 40 mm × 40 mm × 4 mm thermoelectric Peltier cooler. The capillary was placed in contact with the thermoelectric Peltier elements using thermal paste (52022JS, AOS) to ensure uniform heat transfer between both surfaces. We measured the surface temperature of the capillary section in the irradiation zone by using an infrared thermal camera (ICP-9640). The exiting fluid from the irradiation zone was in contact with a 20 mm × 40 mm × 4 mm thermoelectric Peltier element set at 45 °C for the dissolution of the exiting crystals. There was a 10 mm distance between the edges of the Peltier elements where the fluid is exposed to the laser, and crystals were viewed with the microscope.

The urea solution was exposed to a continuous train of 6 ns pulses at a repetition rate of 10 pps from a 1064 nm-wavelength Q-switched Nd:YAG pulsed laser (Continuum Surelite II). The laser beam passed through a 5 mm-diameter ceramic aperture. The beam was linearly polarized by using a Glan Taylor calcite polarizer cube. The incident beam’s average power was measured with a thermopile sensor. The observation zone was located at the center of the irradiation zone or positioned 7.5 mm downstream from the irradiation zone center. Videos were recorded at 60 fps with a 20 MP CMOS camera mounted on an Amscope metallurgical microscope with a 10× objective. The irradiated volume was 5 μL. Each volume element was exposed to an average of 10 laser pulses, at a fluid flow rate of 300 μL/min and a laser repetition rate of 10 pps. During some laser-induced bubble experiments, a Teflon AF dual-layer membrane degasser (Model 403, Vici Metronics) was connected in the flow path between the feed bottle and the pump.

### 2.5 Hydrophobic Treatment of the Glass Capillary

The glass capillary was treated with octadecyltrichlorosilane (95%, Thermo Scientific Chemicals). The process was conducted under a fume hood. A fresh, clean glass capillary was placed in a flat-bottomed glass test tube. A mixture of 10 mL of toluene, 2.5 mL of chloroform, and 25 μL of octadecyltrichlorosilane (OTS) was prepared, swirled gently, and quickly transferred to the test tube containing the capillary. The test tube was capped and left undisturbed for 1 h. The capillary was then rinsed at least 3 times with a 50:50 vol mixture of toluene and chloroform.^23^ The capillary was placed on a wide watch glass and moved to a vacuum oven at 110 °C for overnight baking. After heat treatment, the capillary was checked for uniform coating by manual water droplet contact angle tests.

## 3 Results and Discussion

### 3.1 Critical Supersaturation Determination

Obtaining the limit of supersaturation for the solution system is vital to narrowing down the starting conditions for NPLIN experiments. Supersaturation, *S*, is defined as the ratio of the solution concentration to the concentration of a saturated solution at a given temperature.

Preliminary experiments began in batch glass vials. Thirty glass vials per supersaturation were each filled with 3 mL of supersaturated, unfiltered, undoped solution *S* = 0.95, 1.1, 1.2, 1.3, 1.4, 1.5, and 2.0 with respect to 20.7 °C. For preliminary experiments, the temperature of 20.7 °C was chosen as the reference temperature as the solubility at that temperature is known from published literature.^22^ All transfers were done while the solution was warm and with preheated syringes, needles, and glass vials. The glass vials were then left undisturbed for 24 h and observed afterward. All vials of *S* = 0.95, 1.1, 1.2, 1.3, and 1.4 remained stable without crystals present after 24 h; the vials were observed with the naked eye. However, most of the *S* = 1.5 vials (22/30) spontaneously nucleated, and all of the *S* = 2.0 vials (30/30) nucleated after 24 h. Interestingly, we note that *S* = 1.5 and 2.0 vials had a mix of small prismoid and needle crystals. We moved forward with laser tests for *S* = 1.3, 1.4, and 1.5 solutions at 250 MW/cm^2^ for 1 min of irradiation. The laser was positioned at the center of the solution height for each vial. Each vial was then inspected 2 h after laser irradiation. The results of the laser tests are presented in Figure 2. The control vials in these tests were not exposed to the laser but were placed in a spot for irradiation. The results were inconclusive; we were unable to distinguish the contributing effect of the laser to nucleation. Mechanical agitation by moving the vials was likely responsible for nucleation as the solutions were sensitive to agitation and prone to nucleating easily.

**Figure 2:**
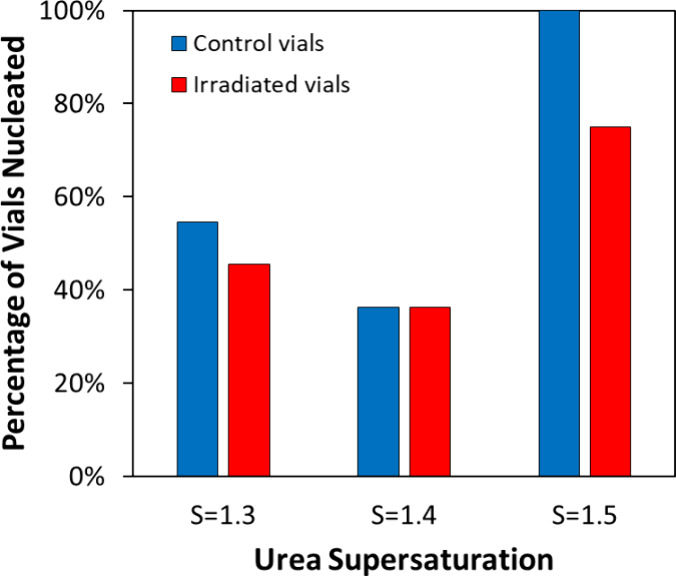
Results of laser experiments with batch glass vials for 3 supersaturations, *S* = 1.3, 1.4, and 1.5. Ten vials were irradiated per supersaturation, and 10 vials were kept as control per supersaturation. Each glass vial was exposed to a laser intensity of 250 MW/cm^2^ for 1 min (red bars). Control vials (blue bars) were not exposed to the laser but were moved to and from the irradiation spot.

We prototyped and 3D printed a vial holder, as shown in the Supporting Information, to minimize the handling of the vials before and after laser exposure. Five solutions (*S* = 1.0, 1.1, 1.2, 1.3, 1.4 with respect to 25 °C) were prepared. 3 mL volumes were transferred into 10 glass vials per supersaturation. A vial holder was used to hold 10 vials per supersaturation and kept at 60 °C for 2 days for dissolution before being held at 25 °C in the oven for 2 days of aging before observation. Here, 25 °C was the lower limit of the convection oven, and lower temperatures could not be set. The vial holders were held by their handles, and each vial was inspected by the naked eye for any crystals. No crystals were noted in all vials after each supersaturation. Although solutions of *S* = 1.4 did not nucleate while undisturbed, handling by directly touching the glass vials of *S* = 1.4 solutions nucleated the solutions within 5–10 min.

Considering the sensitive nature of the urea solutions in batch glass vials, we continued experiments with the flow system. The microfluidic system is not externally agitated and is not subjected to perturbations during the continuous delivery and irradiation of the solution. This would rule out the likelihood of mechanical-shock-induced nucleation that could occur with batch glass vials during handling.

A supersaturated solution of *S* = 1.3 with respect to 25 °C (2.05 g of urea in 50 g of isopropanol) was prepared and connected to the pump inlet tubing while warm. The solution was not aged before use. At a flow rate of 300 μL/min, the cold Peltier was lowered in a stepwise manner with a step of 5 °C (as shown in Table 1) from 25 to −5 °C; a hold of 10 min at each set temperature was given. The solution was not exposed to the laser.

**Table 1:** Measured Temperatures of the Capillary Surface Outside of the Cooled Zone Where the Laser Interacts with the Supersaturated Urea–Isopropanol Solution Flowing at 300 μL/min at a Given Cold Peltier Surface Temperature^a^

cold Peltier temp (°C)	measured temp (°C)	estimated supersaturation
25	24.60	1.3
20	21.10	1.4
15	16.77	1.6
10	13.63	1.7
5	10.23	1.9
0	6.89	2.0
–5	3.76	2.2

a Supersaturation values were estimated with respect to the measured temperatures.

When the cold Peltier surface was set at −5 °C, giving a supersaturation of *S* = 2.2, needle crystals crashed out of the solution and eventually clogged the capillary as shown in Figure 3. No crystals were observed for S < 2.2. The solution concentrations at the measured temperatures were estimated based on reported solubility data.^22^ We determined that a supersaturation of 2.2 is the critical supersaturation or supersaturation limit for our system.

**Figure 3:**
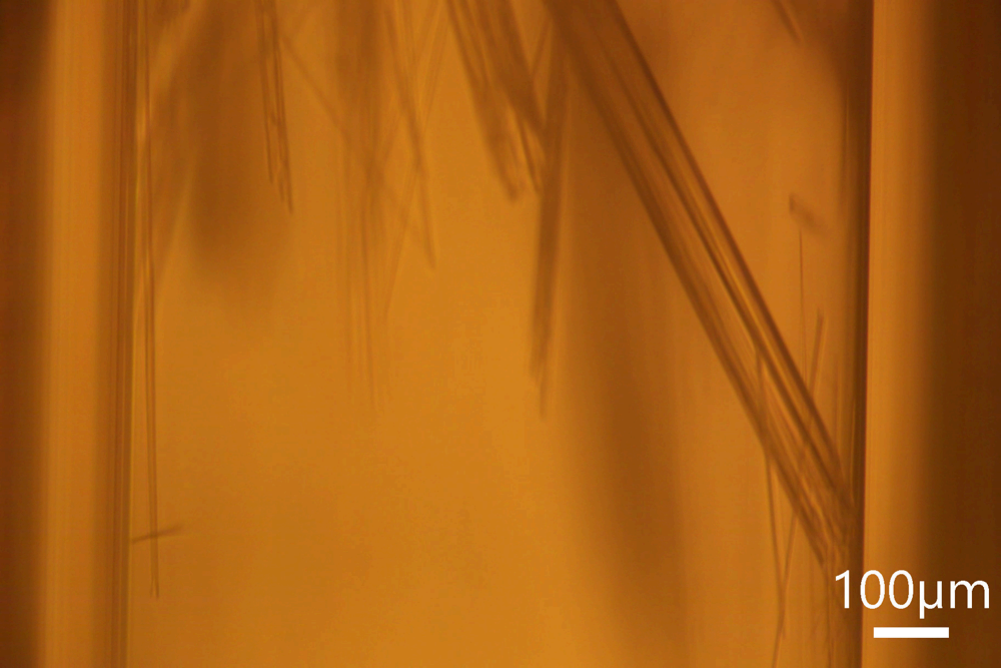
Snapshot of needle urea crystals in isopropanol during flow experiments for determining critical supersaturation. The cold Peltier was set at −5 °C, flow rate of 300 μL/min, no laser exposure, 2.05 g of urea in 50 g of isopropanol prepared in the feed bottle. Scale bar of 100 μm.

### 3.2 Laser-Induced Small Prismoid Crystals

A solution of 2 g of urea in 50 g of isopropanol was prepared, aged for 1 day at room temperature, and then fed into the microfluidic system at 300 μL/min. The cold Peltier was set at 11 °C to give an estimated supersaturation of *S* = 1.65. The solution was exposed to an average laser power of 1.46 W, which corresponded to a laser intensity of 124 MW/cm^2^. A slurry of small crystals (10–30 μm long) was observed in flow during the 3 min irradiation, as shown in Figure 4. These crystals were similar in appearance when compared to the reported prismoid crystal habit in isopropanol.^21^ A control run of 3 min, where the solution was not exposed to the laser, resulted in no crystals. For these experiments, the microscope objective was positioned 7.5 mm downstream from the center of the irradiation zone.

**Figure 4:**
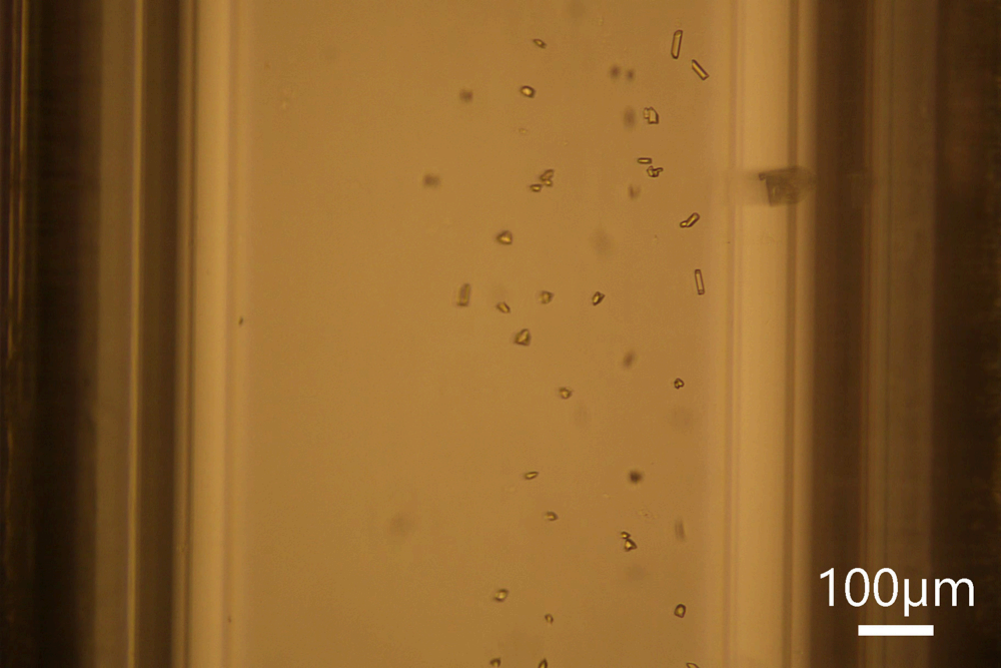
Snapshot of small prismoid urea crystals in isopropanoll during laser exposure. The cold Peltier was set at 11 °C, flow rate of 300 μL/min, 2 g of urea in 50 g of isopropanol prepared, and 1-day aged. Scale bar of 100 μm.

However, subsequent trials with the conditions described here often clogged the pump flow path originating from crystal nucleation in the feed bottle. These disruptions resulted in significant downtime for troubleshooting and declogging. Lower supersaturated solutions fed into the pump could address this concern as the solutions would be less prone to nucleating.

### 3.3 Laser-Induced Bubbles

Unexpectedly, during some laser experiments, we observed a few microbubbles moving with the crystals. Also, during rinses with ultrapure water and with the laser running, bubbles would nucleate from the walls (as shown in Figure 5), but the number count gradually reduced over a 1 h rinse. We referred to this observation as laser-induced degassing. We suspected that the difference in air solubility in the water and isopropanol might be contributing to this observation with the laser. For instance, the solubility of nitrogen in isopropanol is 0.038 M compared with that in water, which is 0.0025 M.^24^ As water and isopropanol mix, bubbles may nucleate, analogous to how solids nucleate with antisolvent addition.^25^ In our work, the laser might accelerate the bubble nucleation process. To further investigate, we intentionally bubbled air in separate bottles of ultrapure water and isopropanol for 30 min. Bottles of ultrapure water and isopropanol were also sonicated for 30 min to compare as a control; sonication is routinely used to degas solvents. The solvents were not mixed; each solvent was separately injected into a dry capillary. Both solvent sets (aerated and sonicated) were irradiated at 155 MW/cm^2^ in flow, but no microbubbles were observed in either set. We concluded that impurity particles might be responsible for the observation of these microbubbles. These impurities might have originated from the urea reagent or dust particles in ambient air.

**Figure 5:**
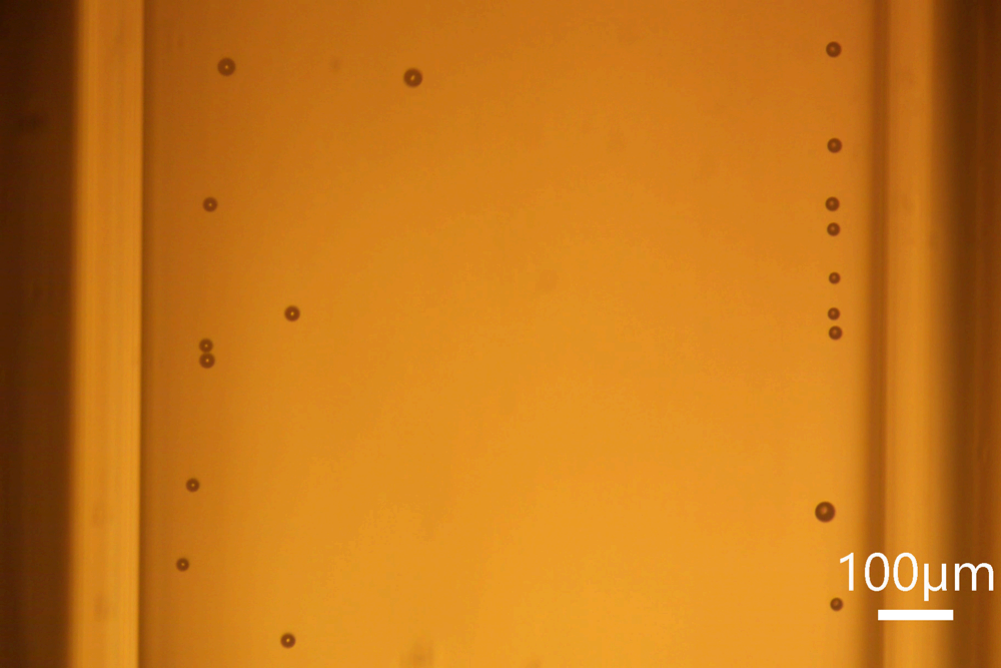
Bubbles nucleated by the laser at 155 MW/cm^2^ during water rinsing of the glass capillary previously containing only isopropanol. Water flow rate: 300 μL/min. Scale bar of 100 μm.

After more scrutiny, we suspected that dust particles played a role. Dust particles could deposit in the feed bottle during the change of solvents for rinsing. Dust particles are typically carbonaceous and strongly absorb light with a wavelength of 1064 nm.^26^ For a controlled experiment, we tested doped solutions. A solution of 1.35 g of urea in 50 g of isopropanol (*S* = 0.93 with respect to 21.5 °C) was doped for an estimated nanoparticle concentration of 10^12^ Fe_3_O_4_ nanoparticles/mL. Nanoparticle characterization was performed with transmission electron microscopy (TEM) to confirm an average particle diameter of 14.8 ± 4.3 nm. See the Supporting Information for more details about the particle size distribution. The undersaturated solution was prepared to avoid crystal nucleation and isolate the nucleation of bubbles. The doped solution was pumped into the microfluidic system, the cold Peltier set at 21.5 °C, equivalent to room temperature, and the flow was stopped. The microscope was positioned above the irradiation zone, and a single laser pulse with a laser intensity of 155 MW/cm^2^, was shot. As shown in Figure 6, bubbles nucleated immediately and rose to the top of the capillary. The bubbles grew to a maximum diameter of 30–40 μm before some collapsed, and others coalesced to form larger bubbles. The few remaining bubbles could be stable for at least 1 h, which implies that they are gas-filled bubbles.

**Figure 6:**
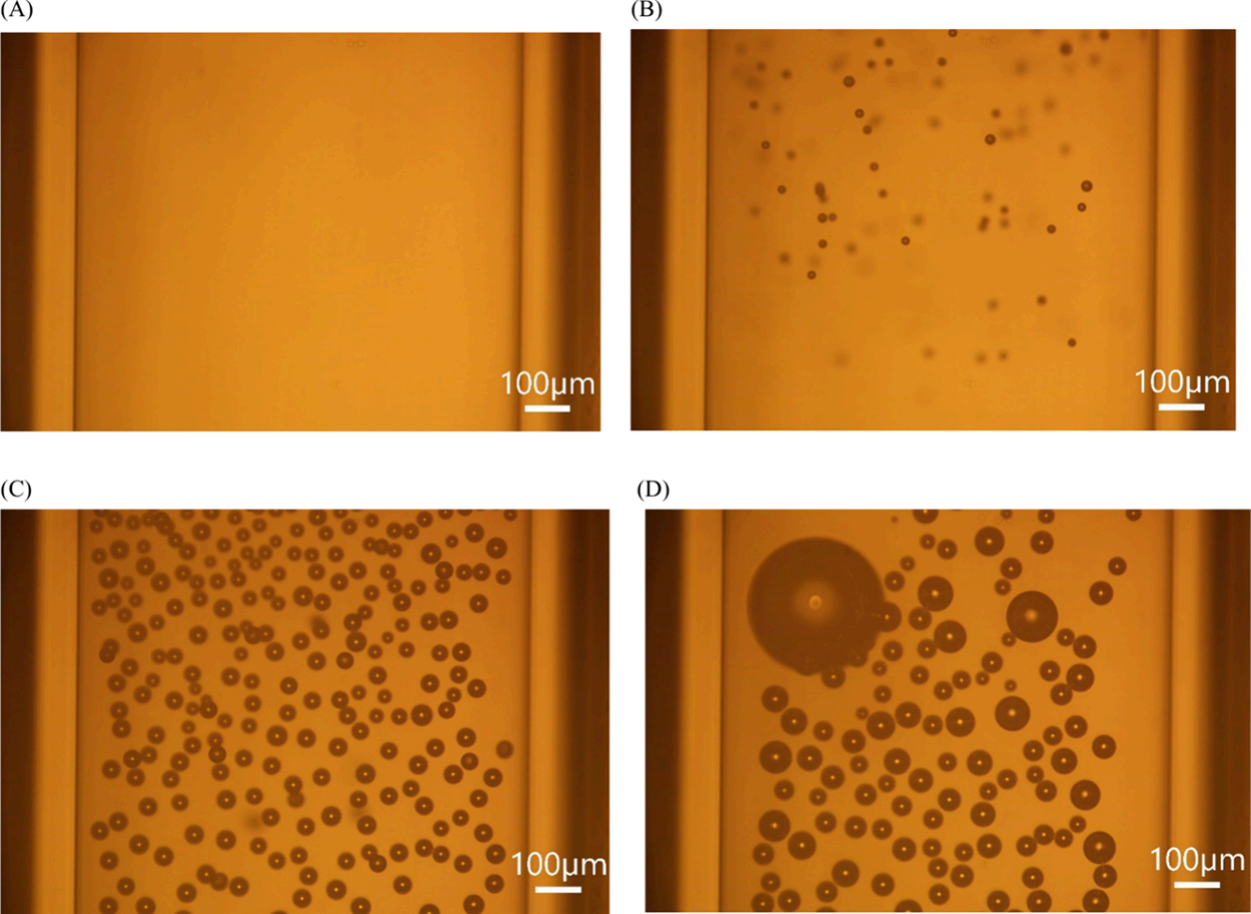
Snapshots of a stationary fluid of *S* = 0.93 and doped with 10^12^ Fe_3_O_4_ nanoparticles/mL exposed to a single laser pulse of 155 MW/cm^2^. (A) Before the laser pulse. (B) 0.5 s after the laser pulse. (C) At *t* = 3 s after the laser pulse, bubbles grow to maximum size. (D) At *t* = 18 s after the laser pulse, some bubbles collapse, and others coalesce. Scale bar of 100 μm.

The nanoparticle concentration was reduced to levels comparable to those in our previous work.^18^ Volumes of 0.5, 1.0, and 1.5 μL of the 4.4 mg/mL Fe_3_O_4_ dispersion were added to 50 mL of filtered solutions of *S* = 0.93 to give estimated nanoparticle concentrations of 3 × 10^9^, 6 × 10^9^, and 9 × 10^9^ nanoparticles/mL, respectively. The flow rate was set at 300 μL/min and at a laser intensity of 155 MW/cm^2^. Bubbles nucleated in the flow and moved with the solution. The average bubble diameters were 19 ± 11 μm, 19 ± 8 μm, and 19 ± 9 μm for the solutions doped with 3 × 10^9^, 6 × 10^9^, and 9 × 10^9^ nanoparticles/mL, respectively. Figures 7 and 8 show the bubble size distribution and the number count from the flow experiments, respectively.

**Figure 7:**
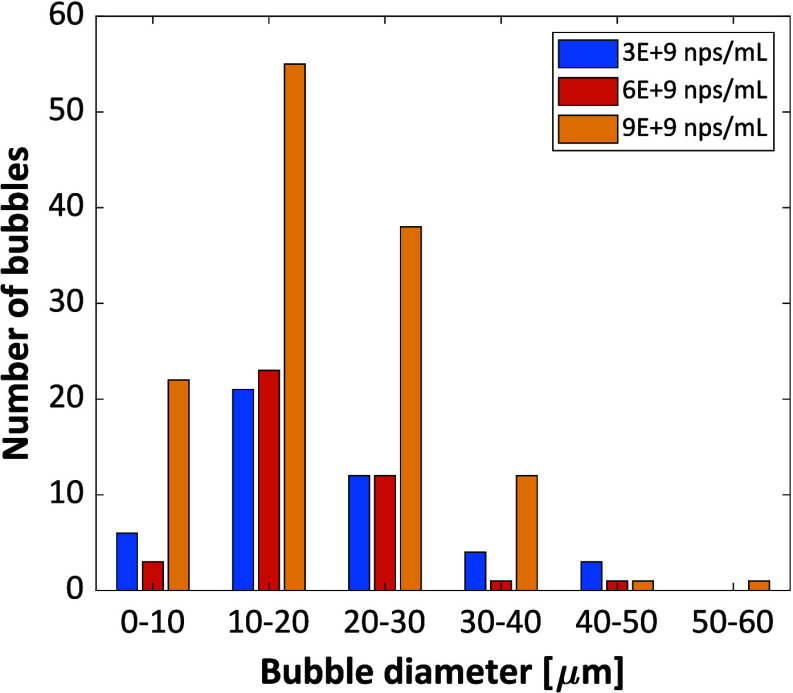
Bubble size distribution from flow experiments. 300 μL volumes of Fe_3_O_4_ nanoparticle-doped urea–isopropanol solutions of *S* = 0.93 exposed to a laser intensity of 155 MW/cm^2^.

**Figure 8:**
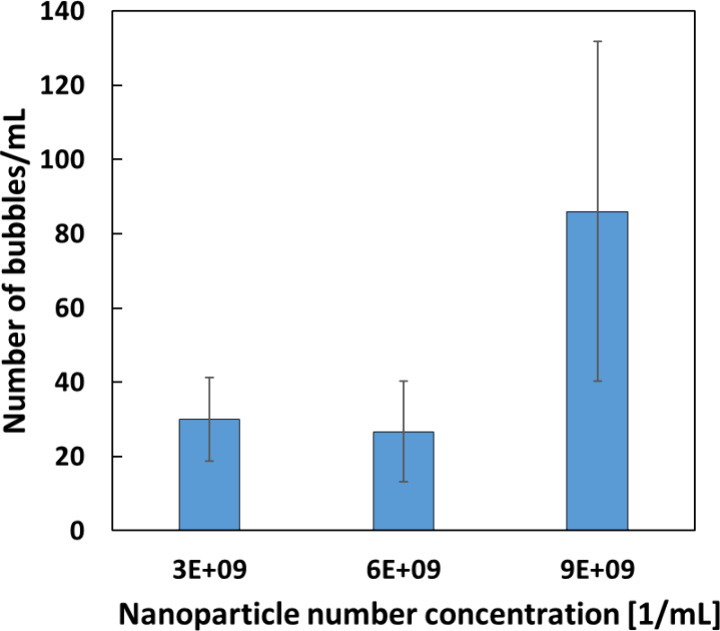
Number of laser-induced bubbles observed per mL of solution irradiated vs nanoparticle number concentration without inline degassing. 300 μL volumes of Fe_3_O_4_ nanoparticle-doped urea–isopropanol solutions of *S* = 0.93 exposed to a laser intensity of 155 MW/cm^2^.

If the dissolved air content in the solution was responsible for the observed microbubbles, then eliminating the dissolved gas content in the solution before irradiation should result in no laser-induced bubbles. Inline vacuum degassing has been used in microfluidics to remove gaseous byproducts.^27,25^ We observed that including an inline vacuum degasser in the flow path before the pump inlet resulted in a near-zero count of the number of laser-induced bubbles. The inline degasser used in this work is rated to remove 94% of air at a flow rate of 1 mL/min. The experimental layout with the degasser is shown in the Supporting Information. Also, without the degasser, applying a backpressure of 750 psi (52 bar) to the system resulted in no laser-induced microbubbles; all other parameters were kept constant. Under elevated pressures, bubble formation will be suppressed, because it is more difficult for the liquid to vaporize or for the bubble to expand.

Previous studies^7,26^ demonstrated that carbon dioxide bubbles can be nucleated in water supersaturated with CO_2_ exposed to nanosecond laser pulses. Ward et al.^26^ found that filtering the solutions reduced the number of bubbles and attributed this observation to extrinsic particles, likely nanoimpurities. In our work, the solution is saturated with air, but the addition of iron oxide nanoparticles resulted in the laser-induced nucleation of gas bubbles. This suggests that supersaturation of air is generated locally around the heated nanoparticle for nucleation of the gas bubble.

A recent study by Barber and Alexander showed, with high-speed imaging, that cavitation bubbles precede cesium crystal nucleation under NPLIN conditions.^12^ Additionally, in a study on laser-induced nucleation of sodium acetate with polymer additives,^28^ only stable bubbles were observed at high polymer additive concentrations. Barber et al. observed that stable bubbles nucleated alongside crystals and proposed that the nucleation of bubbles was due to either thermochemical reactions that produced gas products or nucleation of air that was dissolved in the solution during preparation. Both studies suggest that the dissolved gas may play a role in NPLIN. However, the pathway to crystal nucleation from the gas bubbles is still unknown.

Using focused laser beams, cavitation bubbles can be generated in solutions doped with soluble impurities.^29,30^ Within microseconds, micrometer-sized vapor bubbles formed at the focal point of the laser. The bubble expands, shrinks, and collapses, leaving behind small gas bubbles that persist minutes later with crystals present. Cavity growth and collapse were noted to occur within about 200 μs. This could explain a likely pathway for the observation of gas bubbles in our experiments. As highlighted in Figure 6, the gas bubbles were apparent within 0.5 s after the laser pulse, which is sufficient time for a vapor bubble to have collapsed and any secondary gas bubbles to remain and be captured with microscope resolution. In this work, capturing evidence of vapor bubbles would be difficult due to their nanometer sizes.

The observation of microbubbles within the irradiation zone suggests that the nanoparticles dispersed in the solution interact with the laser energy. The doped-solution experiment revealed that the microbubbles observed could be directly associated with the laser’s interaction with nanoparticles that strongly absorb light at 1064 nm. According to the framework for bubble size estimations described by Ward et al.,^26^ the absorbed energy goes into vaporizing the surrounding solvent, and a vapor bubble forms. The formation of a vapor bubble at the nanometer scale would occur in a degassed solution, assuming that only the liquid solvent undergoes a phase change to vapor.

Using the methodology outlined by Ward et al.,^26^ the vapor bubble size was estimated for a single Fe_3_O_4_ particle in isopropanol exposed to a single laser pulse. The details of the calculations are provided in the Supporting Information. We estimated that a 347 nm-diameter vapor bubble would form from a 15 nm-diameter Fe_3_O_4_ particle exposed to a laser intensity of 155 MW/cm^2^. Although nanobubbles are predicted for the parameters used in this work, the nanobubbles likely grew to micrometer sizes due to the incorporation of gas molecules previously dissolved in the solution.^31^

For a nondegassed solution, with a sufficient concentration of dissolved gas capable of enlarging the nucleated vapor bubble, gas molecules in the surrounding solution would diffuse across the bubble interface due to a concentration gradient. The resulting bubble size would be larger for the nondegassed solution compared to the degassed solution. The difference is dependent on the concentration of dissolved gas in the solution. Also, very few bubble nucleation events are likely to occur compared to the nanoparticle concentration. For instance, from Figure 7C, the number of bubbles is 223. The volume of solution captured in the image is about 0.9 μL, which corresponds to 9 × 10^8^ nanoparticles for the given volume. For a laser intensity of 155 MW/cm^2^, the ratio of bubbles to nanoparticles is 2.5 × 10^–7^. This means that the fraction of irradiated nanoparticles that give rise to gas bubbles is extremely low.

Another possible route for gas microbubble formation is nucleation at the vapor bubble/solution interface. Applying a simple geometrical argument,^32^ we can estimate the increase in the gas concentration caused by a vapor bubble. Here, we assume that the radius of the induced vapor bubble is larger than that of the nanoparticle and that the gaseous solute (air), previously within the volume occupied by the vapor bubble, is swept up to the bubble interface through diffusion where it is confined to a spherical shell surrounding the bubble. Solute molecules would also diffuse away from the interface over time, which affects the shell thickness. The spherical shell surrounding the vapor bubble with thickness δ can be approximated by ${\left(Dt\right)}^{1/2}$ , where *D* is the solute diffusion coefficient and *t* is the time. For instance, the diffusion coefficient of oxygen in isopropanol^33^ at 25 °C is 3.3 × 10^9^ m^2^/s. From simulations,^34^ supersaturation peaks at the bubble interface at 1 μs. For oxygen gas, we calculated the shell thickness, δ = 57 nm. Using the isopropanol vapor bubble radius of 173.5 nm, the oxygen gas concentration at the bubble interface increases by a factor of 1.7. Such an increase in local concentration could trigger the nucleation of an oxygen gas bubble. Here, the vapor bubble is assumed to have survived for a few microseconds, which would be sufficient time for a concentrated shell of dissolved oxygen molecules at the bubble–solution interface to remain and for nucleation to be triggered. Our observations show that upon laser exposure, the dissolved air content in a prepared urea–isopropanol solution can be liberated in the form of micron-sized bubbles.

### 3.4 Laser-Induced Needle Urea Crystals

During this study, we observed the transformation of the needle crystals into smaller aspect-ratio crystals. A solution of 2.05 g of urea in 50 g of isopropanol was prepared in a glass bottle and left to age at room temperature (21.5 °C) for 2 days for an estimated supersaturation of *S* = 1.4. Crystals nucleated spontaneously, and needles grew and subsequently transformed into smaller prismoid crystals after gently swirling the bottle by hand. All needle crystals had transformed into smaller prismoid crystals after 10 min of intermittent swirling of the solution. This suggests that at sufficiently high supersaturations (*S* ≥ 1.4), the kinetically favored crystal habits in the form of needle crystals grow first and transform into a smaller prismoid habit as the supersaturation decreases.

Here, we report conditions in which the laser induces the nucleation of urea crystals in isopropanol. Needle crystals grew and were observed. A solution of 1.73 g urea in 50 g isopropanol (*S* = 1.2 with respect to 21.5 °C) was filtered with a 220 nm pore nylon syringe filter. 1 μL of 0.44 mg/mL Fe_3_O_4_ nanoparticle dispersion was added to give an estimated nanoparticle concentration of 10^9^ nanoparticles/mL. The feed bottle was placed on a hot plate-stirrer set to give a liquid temperature of 40 °C and 100 rpm with a magnetic stirrer bar to keep the solution mixed and warm during injection. The solution was not aged. The cold Peltier was set at −13 °C, and the measured temperature just outside the cooled zone was −0.64 °C to give a supersaturation of 2.1. Crystals nucleated at a threshold laser intensity of 158 MW/cm^2^. The same conditions were repeated but with a higher nanoparticle concentration of 10^10^ nanoparticles/mL (added 1 μL of 4.4 mg/mL Fe_3_O_4_ nanoparticle dispersion). A slightly lower laser intensity threshold of 153 MW/cm^2^ was noted.

We captured the transformation of needle crystals to prismoid crystals. The glass capillary surface was hydrophobized to help mitigate flowability challenges, see the Supporting Information for more details. During some trials, needle crystals clog the capillary. The flow was stopped to allow the transformation to occur slowly. As shown in Figure 10, at time = 0 min, mostly needle crystals are present in the capillary section. After 5 min, the needle crystals dissolved into the solution, and the prismoid crystals grew larger. We suspect that the needle crystals grow at high supersaturations, and the small prismoid crystals are the kinetically preferred habit at lower supersaturations. When the flow was stopped, the fluid began to warm up from −0.64 °C to room temperature (21.5 °C) during the 5 min period, which permits the dissolution of the needle crystal habit and fosters the growth of the smaller prismoidal crystal habit. As the solution temperature increases, the supersaturation decreases, assuming that the solute concentration is kept constant, although the solute concentration is also decreasing as the crystals grow. The crystal habit depends on the growth rates of the different crystallographic faces.^35^ For reference, urea’s crystal faces are depicted in Figure 9. For instance, in water and at *S* = 1.0034, the estimated growth rate of the {110} face is between 0.001 and 0.01 μm/s, and for the {001} face, the experimental growth rate in water is about 0.3 to 0.5 μm/s.^36^ The needle habit appears due to the faster growth of the {001} face.

**Figure 9:**
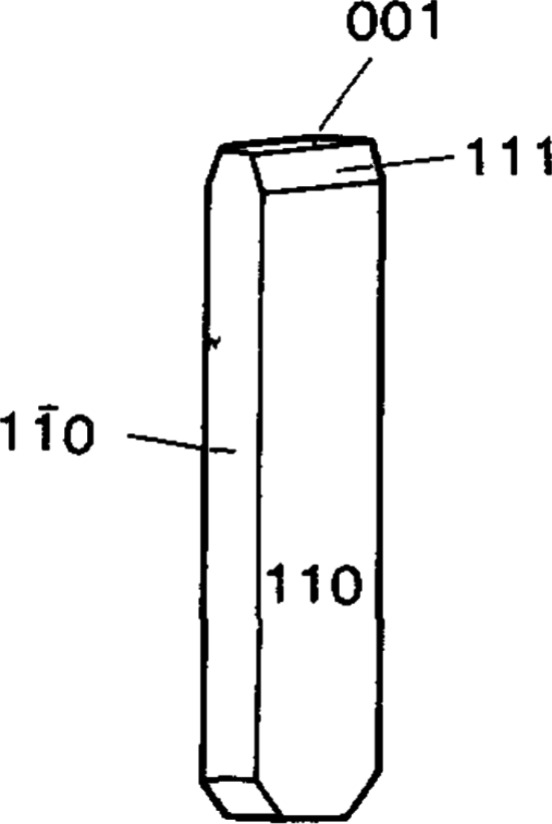
Urea crystal habit in aqueous solutions.^36^

The image was reproduced with permission from ref 36. Copyright 1986 Elsevier.

Hodorowicz and Treivus studied the growth kinetics of a urea crystal’s {001} face across a subcooling range from 0.01 to 0.91 °C in aqueous solution.^37^ A noticeable increase in the growth rate was reported at higher supersaturations. For instance, at a subcooling of 0.01 °C, the mean growth rate was 1.6 × 10^–6^ cm/s, and at a subcooling of 0.21 °C, the mean growth rate was 30.1 × 10^–6^ cm/s.^37^ We speculate that in isopropanol at high supersaturations, the {001} face is growing much faster, analogous to the behavior in water. This could explain the observation of needle crystals in isopropanol initially before transformation.

**Figure 10:**
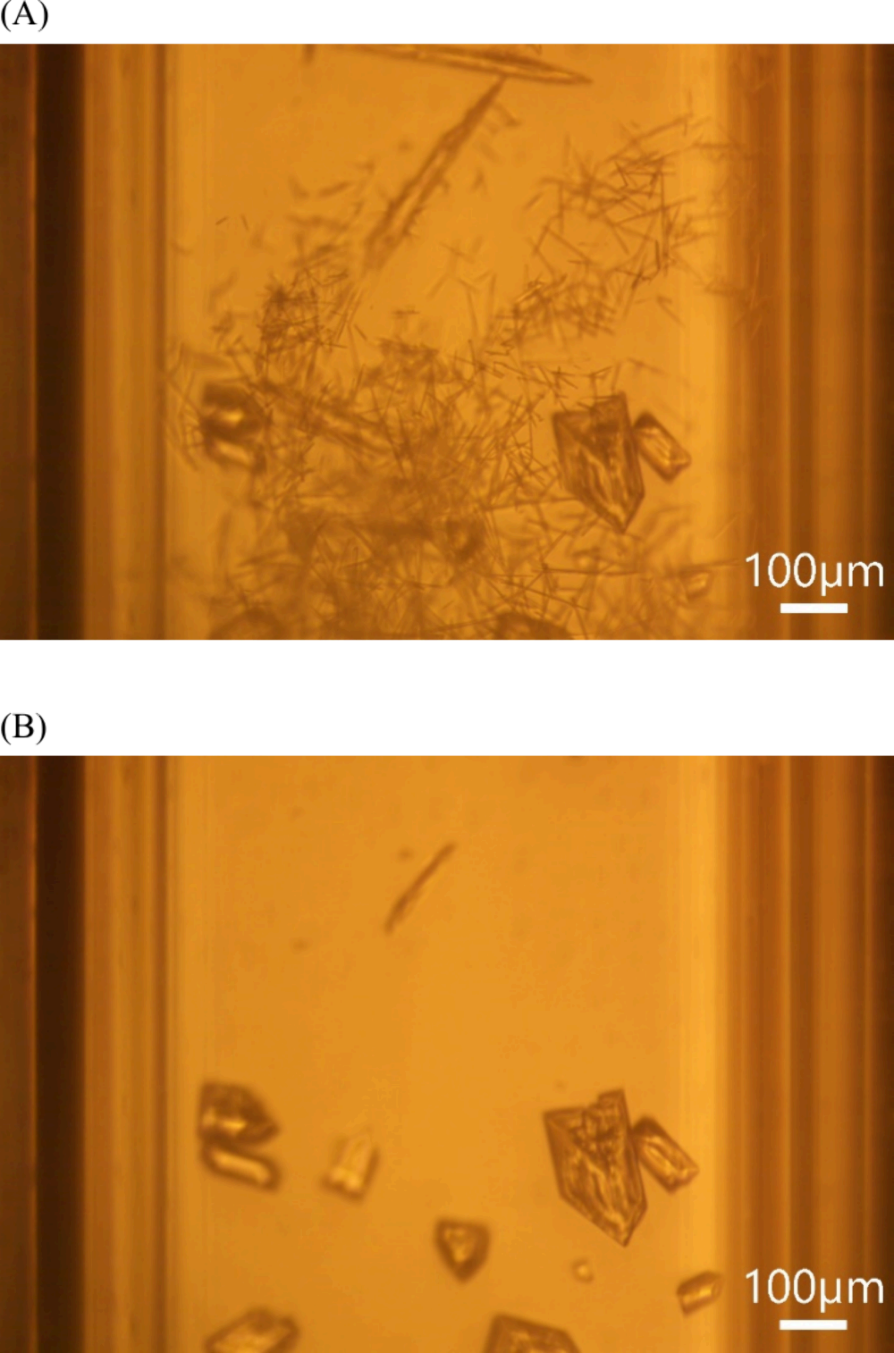
Snapshots from a video of the transformation of needle urea crystals into prismoid (smaller aspect ratio) crystals at (A) time = 0 min. (B) After 5 min. Scale bar of 100 μm.

In isopropanol, the urea crystal is more compact and has a reported aspect ratio of 1.44.^21^ Using the shape diagram from Salvalaglio et al.,^38^ we can estimate that the ratio of the growth rates of the {110} face to {001} face is 0.14 to 1, and the ratio of the growth rates of the {111} face to {001} face is 0.17 to 1. In isopropanol, the 3 dominant faces would be growing at comparable rates, hence the observed compact crystal habit over time.

Only one polymorph of urea has been reported for ambient pressures.^39^ In the discussion above, we assumed that both the urea needles and prismoidal crystals consist of this polymorph. There is a remote possibility that the needles consist of a metastable polymorph or solvate that transforms into the stable polymorph; although given the ubiquitous needle shape of the stable form of urea, we believe this is unlikely.

## 4 Conclusions

In summary, we used microfluidics to determine the critical supersaturation of the system to be *S* = 2.2, where crystals spontaneously nucleated in flow, and needle crystals were observed. Smaller aspect-ratio prismoid crystals were observed downstream from the laser exposure zone using prepared supersaturated solutions cooled to a supersaturation of *S* = 1.65. We noted that a few microbubbles were moving with the crystals and found that microbubbles can be readily generated in the urea–isopropanol solutions by the addition of iron­(II, III) oxide nanoparticles. From a stationary no-flow experiment, the laser-induced bubbles grew to a maximum diameter of 30–40 μm. In flow experiments, the bubbles have an average diameter of 19 μm. Using an inline degasser during the experiments, we were able to confirm that the bubbles were gas-filled bubbles. We estimated a vapor bubble diameter of 347 nm for a 15 nm-diameter Fe_3_O_4_ nanoparticle exposed to a laser intensity of 155 MW/cm^2^. This suggests that there is a fast growth of the bubbles to micrometer sizes due to the dissolved air in the solution. Laser-induced needle crystals were observed at a supersaturation of 2.1. The capillary was treated with octadecyltrichlorosilane to create a hydrophobic surface and help mitigate the bridging of the flow with needle crystals. Needle crystals were observed to transform to smaller aspect-ratio prismoid crystals as the solution warmed to room temperature. This suggests that needle crystals are the preferred habit at high supersaturations in urea–isopropanol solutions. Further optimization of flow and NPLIN conditions can facilitate more thorough probing of the urea–isopropanol solution system, resulting in tunable control of conditions for the nucleation and flow of needle crystals. The findings presented here provide additional support for the impurity-heating mechanism of NPLIN. The observation of laser-induced microbubbles supports the pathway dictated by the impurity-heating mechanism. The observation of needle urea crystals in isopropanol, although unexpected, can guide researchers toward optimal conditions for future urea crystallization studies.

## Supplementary Material



## Notes

Image of glass vial holder, inline degassing setup, nanoparticle size distribution, hydrophobization of the glass capillary, nanobubble size estimation, urea in methanol (PDF)

## †

Department of Chemical and Biomolecular Engineering, University of Pennsylvania School of Engineering and Applied Science, Philadelphia, Pennsylvania 19104, United States (P.H.)

## ABBREVIATIONS

NPLIN: nonphotochemical laser-induced nucleation
OKE: optical Kerr effect
DP: dielectric polarization
IH: impurity-heating
PEEK: polyether ether ketone
ABS: acrylonitrile butadiene styrene
OTS: octadecyltrichlorosilane
TEM: transmission electron microscopy
