# Supplementary material for: Microfluidic Laser-Induced Nucleation of Air Microbubbles and Crystals in Urea–Isopropanol Solutions
Source: Cryst Growth Des. 2026 Apr 3;26(9):3378–87. doi: 10.1021/acs.cgd.5c01702 (PMC13154137; doi:10.1021/acs.cgd.5c01702)
Supplement: Supplementary file 1 [file cg5c01702_si_001.pdf]

Supporting Information

Microfluidic Laser-Induced Nucleation of Air Microbubbles and Crystals in Urea-Isopropanol Solutions

Kelechi F. Ndukwe-Ajala, Pierce Haider, Jasmin M. Sabirin, Jiamu Guo, Bruce A. Garetz*, Ryan L. Hartman*

Department of Chemical and Biomolecular Engineering, NYU Tandon School of Engineering, Brooklyn, New York 11201, United States.

1. GLASS VIAL HOLDER

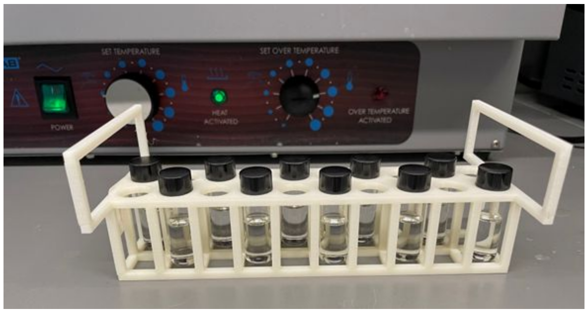

Figure S1. A 3-D printed vial holder made of Acrylonitrile Butadiene Styrene (ABS) thermoplastic for a 360° inspection of glass vials containing urea-isopropanol solutions. Handles at the side minimize agitating the glass vials directly during the inspection for crystals.

2. INLINE DEGASSING.

An inline degasser was added to the flow setup between the feed bottle and the pump. The solution was continuously degassed before entering the pump. The inline degasser (Vici Metronics) has a dual-layer semi-permeable membrane made of Teflon AF®, an amorphous fluoropolymer with high gas permeability. The inline degasser was connected to a vacuum pump using the barbed ports.

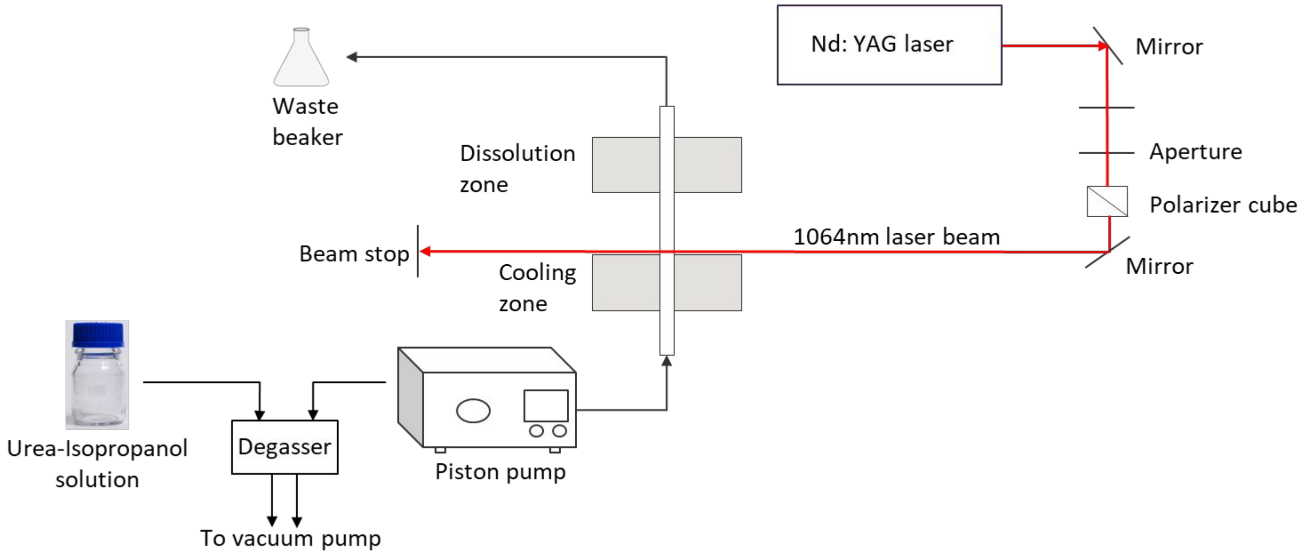

Figure S2. Sketch of experimental setup with inline degasser.

3. NANOPARTICLE SIZE DISTRIBUTION

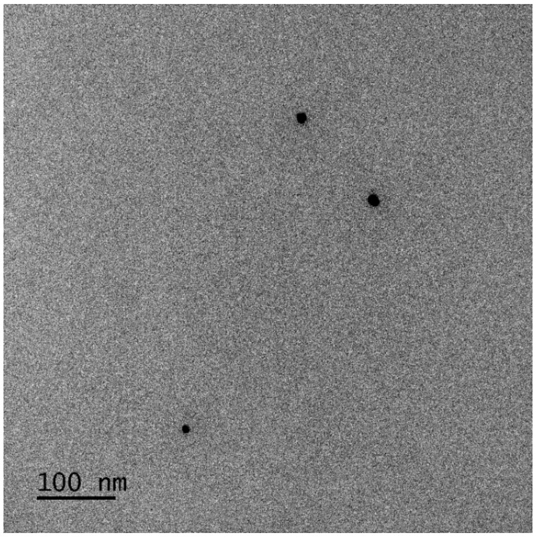

Figure S3. A sample image of iron (ii, iii) oxide nanoparticles. Images were acquired with Transmission Electron Microscopy (TEM).

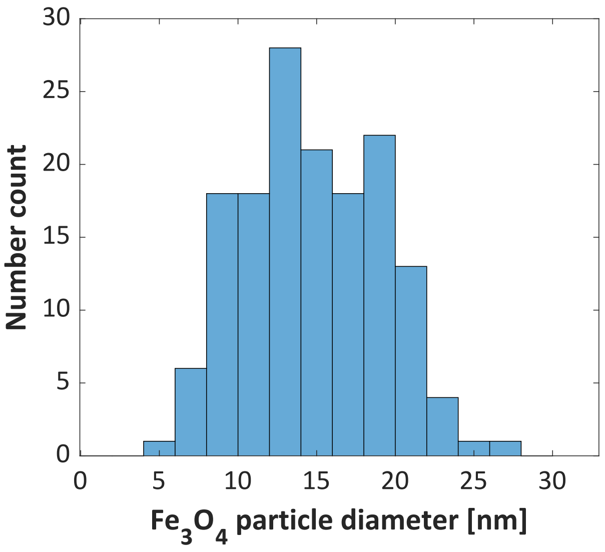

Figure S4. Particle size distribution of iron (ii, iii) oxide nanoparticles. Images were acquired with Transmission Electron Microscopy (TEM), and 151 particles from 3+ images were analyzed. Image analysis was done with ImageJ. Measured average particle diameter of 14.8 $\pm$ 4.3 nm.

4. HYDROPHOBIZATION OF GLASS CAPILLARY

Needle crystals tend to present flowability challenges due to their thin and elongated nature, resulting in hydrodynamic bridging[1] and plugging the capillary. We consistently encountered this clogging issue with the needle crystals. This could be addressed by adjusting the operating conditions, such as flowrate and supersaturation, to reduce the number of needle crystals per time flowing through the capillary space. However, such attempts were not effective as the needle crystals tended to grow fast as they moved in the supersaturated fluid and stuck to the capillary walls. We explored altering the glass wall interactions and changing the surface from hydrophilic to hydrophobic. The glass capillary was treated with octadecyltrichlorosilane. OTS forms a well-ordered methyl-terminated monolayer at the glass surface. This is due to the reaction of the silane compound with trace amounts of water adsorbed on the glass surface.[2] As shown in **Figure S5**, the water meniscus in the OTS-treated capillary is inverted compared to the untreated capillary, highlighting the switch in the wetting behavior of the glass surface. Also, we conducted contact angle measurements with a simple imaging setup of a single water droplet in contact with the outer capillary surface: water contact angle on OTS-treated capillary = 96 $\pm$ 1° and for the untreated capillary = 38 $\pm$ 2°. The OTS treatment partially resolved the issue by allowing the needle crystals to glide off the walls and detach faster with the moving fluid.

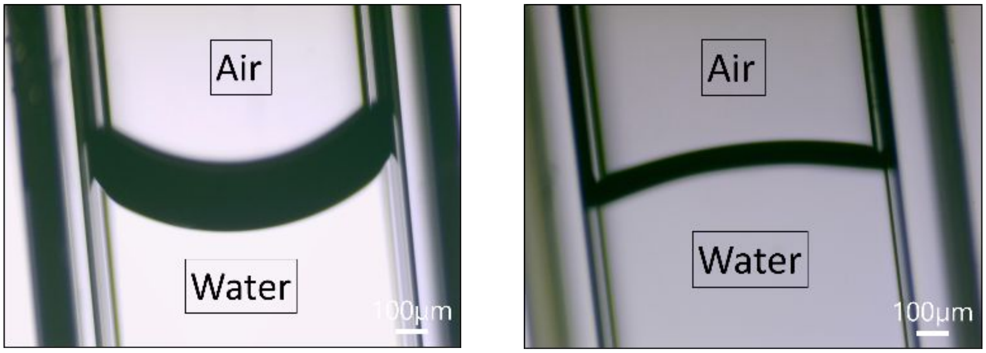

Figure S5. Comparison of the water meniscus in an untreated square glass capillary (left) vs in a square glass capillary treated with octadecyltrichlorosilane (right). Scale bar of 100 µm.

5. NANOBUBBLE SIZE ESTIMATION

As shown by Ward et al.,[3] we can estimate the bubble size from nanoparticle heating. We assumed the surrounding liquid is pure isopropanol with a refractive index n_{ipa} at 1064nm = 1.378.[4] The energy absorbed by a spherical particle of radius a can be estimated by:

$$E_{abs} = \pi a^2 Q_{abs}(a) I t_p, \tag{1}$$

where Q_{abs} is the absorption efficiency. Mie scattering calculations were done using MiePlot v4.6.21 (written by Philip Laven).[5] The complex refractive index of Fe_3O_4 (at 1064nm) is = 2.1112 + 0.3698i.[4] For a = 7.5nm, the Q_{abs}= 0.032. At I = 155 MW/cm^2 and pulse width of t_p = 6 ns, the energy absorbed by the particle E_{abs} = 5.3 × 10^{-14} J.

We assume all the absorbed energy is used to instantaneously heat the isopropanol to T_1 = 406 K (80% of the critical temperature of isopropanol, T_{cr} = 508 K). The quantity of isopropanol that can be heated and vaporized can be estimated by:

$$E_{abs} = n\left(\Delta_{vap} U_{T_1} + \int_{T_0}^{T_1} c_v(T) dT\right), \tag{2}$$

where n is the number of moles and T_0 = 293 K. Data for the heat capacity at constant volume, c_v, for temperatures between 273 and 473 K could not be found in the literature. So ideal gas was assumed to relate c_p and c_v, where c_v = c_p – R. The constant-pressure heat capacity, c_p values were gathered from the literature,[6,7] as shown in **Figure S6**.

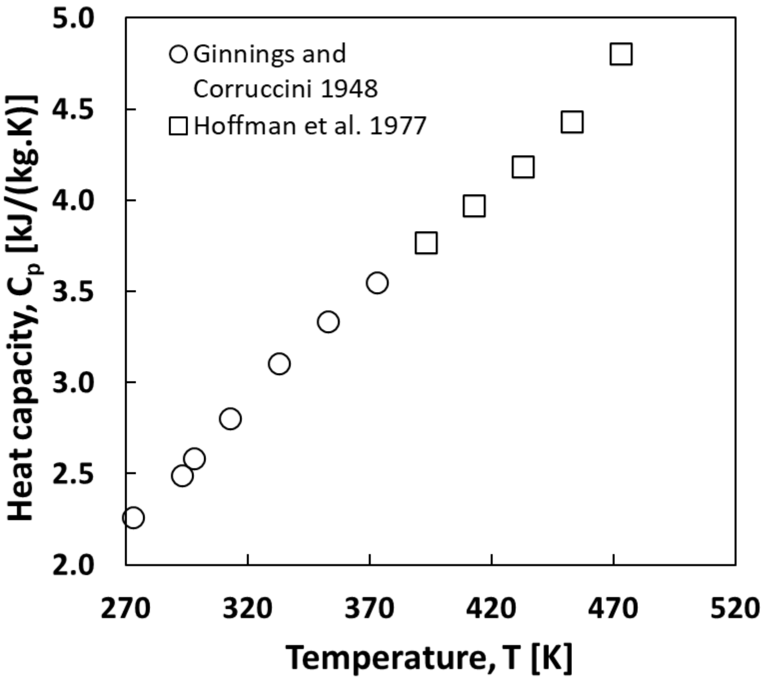

Figure S6. Constant-pressure heat capacity, c_p values of isopropanol gathered from the literature.

The c_v values were then estimated using the ideal gas assumption, as shown in **Figure S7**. The integral was calculated using a linear fit to the c_v data, $\int_{T_0}^{T_1} c_v(T)dT$ = 21.01 kJ/mol . The internal energy change for vaporization = 39.1 kJ/mol (at 410 K).[8] We estimated n = 9.3 × 10^{-19} moles, which gives V_1 = 7.1 × 10^{-23} m^3 at the ambient density of 786 kg/m^3.

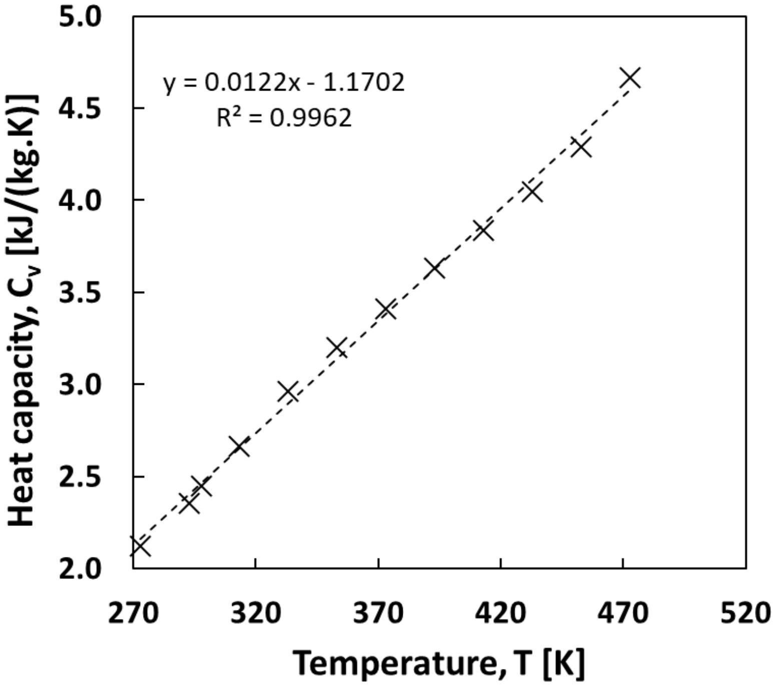

Figure S7. Estimated values of constant-volume heat capacity, c_v of isopropanol.

The size of the resulting bubble was estimated assuming reversible adiabatic expansion. Assuming an ideal gas, pV^{χ} = constant, where $\chi = C_p/C_v$ = 1.06 at 293 K. The initial pressure of the vapor bubble is estimated to be $P_1 = \frac{nRT_1}{V_1}$ = 44 MPa. Assuming a final pressure equal to ambient pressure, P_2 = 101 kPa, the final volume of the bubble, V_2 = 2.2 × 10^{-20} m^3. Combined with the volume of the particle, this gives a final bubble radius of r_2 = 173.5 nm and a diameter of 347 nm.

6. UREA IN METHANOL.

We also studied the NPLIN of urea in methanol. Unexpectedly, during preliminary tests with batch glass vials, we observed thin hexagonal-plate-shaped crystals that we determined were important for laser-induced nucleation of the typical rod-like crystal habit reported for methanol.[9] These thin hexagonal-plate-shaped crystals would form (**Figure S8**), and the solution would appear cloudy; the crystals would settle at the vial bottom after 1 day of aging. If the vials were left undisturbed and not agitated, no transformation into rod-shaped crystals would occur for at least 4 weeks.

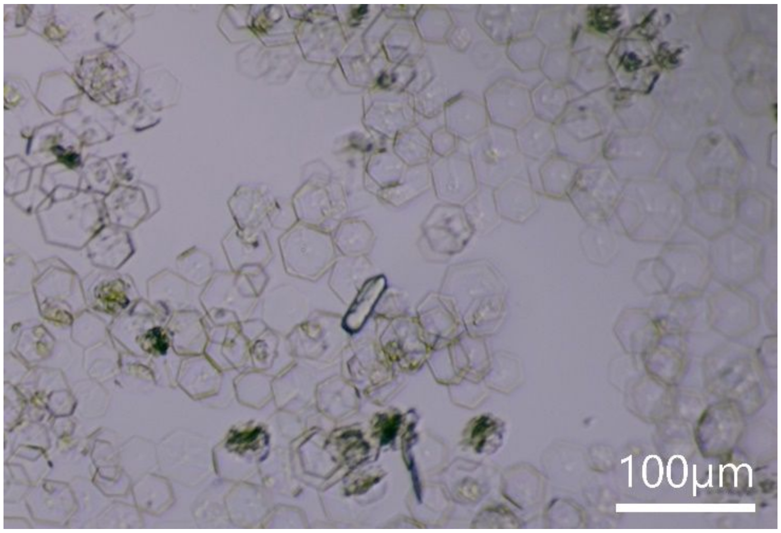

Figure S8. Thin hexagonal-plate-shaped urea crystals with a single typical rod-shaped urea crystal present in a supersaturated $S = 1.5$ urea-methanol solution. Scale bar of 100µm.

When the urea-methanol solutions were exposed to laser pulses, higher nucleation probabilities were observed when the thin hexagonal-plate-shaped crystals were in the laser's path. The solubility of urea in methanol at 20 ^{o}C is 21.79g per 100g methanol.[10] For S=1.5 with respect to 20 ^{o}C solutions aged for 24 hrs, all 7 vials of cloudy solutions exposed to a laser intensity of 142 MW/cm^2 for 1 min nucleated and rod-like crystals appeared during irradiation, compared with 7 vials with the settled thin hexagonal-plate-shaped crystals, where only 2 vials nucleated with rod-like crystals were observed. The laser was positioned at the center of the solution height in the glass vial.

Using a bulb pipette, the mother liquor above the settled thin hexagonal-plate-shaped crystals was carefully extracted to avoid agitation of the glass vial. The methanol in the glass vial was left to evaporate in a fume hood. The glass vial was carefully broken to isolate the thin hexagonal-plate-shaped crystals on the vial bottom. The glass pieces with a thin layer of hexagonal-plate-shaped crystals were transported carefully for X-ray diffraction measurements. However, we were unable to capture reliable offline X-ray diffraction measurements on the thin hexagonal-plate-shaped crystals due to the rapid transformation to the rod-like crystals upon exposure to air and likely evaporation of leftover mother liquor that would lead to a sufficient supersaturation for the transformation. Also, the small isolated quantities (<10 mg) were insufficient for strong diffraction patterns. Isolating the thin hexagonal-plate-shaped crystals in larger quantities ($\geq$ 1g) would provide more reliable measurements and identification of their chemical structure.

Considering that the rod-like crystals are not present prior to laser exposure, we speculate that the solution is supersaturated with respect to the rod-like crystals even with the presence of the hexagonal-plate-shaped crystals. During visual qualitative inspections, we noted that the mass of the plate crystals << mass of rod crystals per given supersaturation per given solution volume. These plate crystals could be a precursor to the rod-like crystals or a metastable phase; more work will be needed to confirm their nature and relevance.

A metastable polymorph would have a higher solubility than the most stable polymorph, the solution could be supersaturated with respect to the stable polymorph, but saturated with respect to the metastable polymorph. For example, in the use of the sodium acetate trihydrate in reusable hand warmers where the aqueous solution is supersaturated with respect to the stable hydrate, but saturated with respect to the metastable anhydrous sodium acetate.[11]

REFERENCES

(1) Hartman, R. L. Managing Solids in Microreactors for the Upstream Continuous Processing of Fine Chemicals. *Org. Process Res. Dev.* **2012**, *16* (5), 870–887. https://doi.org/10.1021/op200348t.

(2) Wong, J. X. H.; Yu, H.-Z. Preparation of Transparent Superhydrophobic Glass Slides: Demonstration of Surface Chemistry Characteristics. *J. Chem. Educ.* **2013**, *90* (9), 1203–1206. https://doi.org/10.1021/ed300809m.

(3) Ward, M. R.; Jamieson, W. J.; Leckey, C. A.; Alexander, A. J. Laser-Induced Nucleation of Carbon Dioxide Bubbles. *J. Chem. Phys.* **2015**, *142* (14), 144501. https://doi.org/10.1063/1.4917022.

(4) Polyanskiy, M. N. Refractiveindex.Info Database of Optical Constants. *Sci Data* **2024**, *11* (1), 94. https://doi.org/10.1038/s41597-023-02898-2.

(5) Laven, P. *MiePlot*. MiePlot. http://www.philiplaven.com/mieplot.htm (accessed 2025-11-14).

(6) Ginnings, D. C.; Corruccini, R. J. Liquid Isopropyl Alcohol - Enthalpy, Entropy, And Specific Heat From 0° To 200° C. *Ind. Eng. Chem.* **1948**, *40* (10), 1990–1991. https://doi.org/10.1021/ie50466a033.

(7) Hoffman, S.; San Jose, J.; Reid, R. Liquid Heat Capacity of Tert-Butyl Alcohol, Isobutyl Alcohol, and Isopropyl Alcohol at High Temperature. *J. Chem. Eng. Data* **1977**, *22* (4), 385–388. https://doi.org/10.1021/je60075a600.

(8) Ambrose, D.; Townsend, R. 681. Thermodynamic Properties of Organic Oxygen Compounds. Part IX. The Critical Properties and Vapour Pressures, above Five Atmospheres, of Six Aliphatic Alcohols. *J. Chem. Soc.* **1963**, No. 0, 3614–3625. https://doi.org/10.1039/JR9630003614.

(9) Shahrir, N.; Nurul'ain Yusop, S.; Anuar, N.; Zaki, H. M.; Tominaga, Y. Influence of Polar Protic Solvents on Urea Morphology: A Combination of Experimental and Molecular Modeling. *Crystal Growth & Design* **2023**, *23* (6), 4240–4254. https://doi.org/10.1021/acs.cgd.3c00060.

(10) Lee, F.-M.; Lahti, L. E. Solubility of Urea in Water-Alcohol Mixtures. *Journal of Chemical and Engineering Data* **1972**, *17* (3), 304–306. https://doi.org/https://doi.org/10.1021/je60054a020.

(11) Barber, E. R.; Ward, M. R.; Alexander, A. J. The Role of Cavitation and Gas Bubbles in the Non-Photochemical Laser-Induced Nucleation of Sodium Acetate. *CrystEngComm* **2024**, *26* (27), 3634–3642. https://doi.org/10.1039/D4CE00487F.
